# Hospital admissions among people who inject opioids following syringe services program implementation

**DOI:** 10.1186/s12954-020-00376-1

**Published:** 2020-05-12

**Authors:** K. J. Bornstein, A. E. Coye, J. E. St. Onge, H. Li, A. Muller, T. S. Bartholomew, H. E. Tookes

**Affiliations:** 1 University of Miami Miller School of Medicine, Miami, USA; 2 Department of Medicine, University of Miami Miller School of Medicine, Miami, USA; 3 Department of Public Health Sciences, University of Miami Miller School of Medicine, Miami, USA; 4 Florida Department of Children and Families Office of Substance Abuse and Mental Health, Tallahassee, USA

**Keywords:** Syringe services program, Take-Home Naloxone, Overdose, Opioid epidemic

## Abstract

**Background:**

Syringe services programs (SSPs) are an evidence-based harm reduction strategy that reduces dangerous sequelae of injection drug use among people who inject drugs (PWID) such as overdose. SSP services include safer injection education and community-based naloxone distribution programs. This study evaluates differences in overdose-associated hospital admissions following the implementation of the first legal SSP in Florida, based in Miami-Dade County.

**Methods:**

We performed a retrospective analysis of hospitalizations for injection drug-related sequelae at a county hospital before and after the implementation of the SSP. An algorithm utilizing ICD-10 codes for opioid use and sequelae was used to identify people who inject opioids (PWIO). Florida Department of Law Enforcement Medical Examiners Commission Report data was used to analyze concurrent overdose death trends in Florida counties.

**Results:**

Over the 25-month study period, 302 PWIO admissions were identified: 146 in the pre-index period vs. 156 in the post-index period. A total of 26 admissions with PWIO overdose were found: 20 pre-index and 6 post-index (*p* = 0.0034).

**Conclusions:**

Declining overdose-associated admissions among PWIO suggests early impacts following SSP implementation. These results indicate a potential early benefit of SSP that should be further explored for its effects on future hospital admission and mortality.

## Introduction

In 2018, the Centers for Disease Control and Prevention announced drug overdose mortality hit a record high, with at least 70,237 Americans dying from an overdose [1]. The impact of the overdose crisis is felt heavily in Florida: opioid-related deaths increased 35% between 2015 and 2016 statewide [2]. Heroin-associated deaths in Miami-Dade County rose 826% between 2011 and 2016 [2]. As Miami-Dade County consistently ranks first in HIV incidence nationwide, implementation of evidence-based HIV prevention coupled with overdose prevention was imperative [3].

In 2016, Florida enacted the *Infectious Disease Elimination Act*, allowing a pilot syringe services program (SSP) but restricted to operate only in Miami, Florida: the University of Miami IDEA SSP. The World Health Organization, the Centers for Disease Control, and the United Nations have found SSPs to be cost-effective in reducing infectious disease burden [4–6]. In the year following the establishment of IDEA in Miami, approximately 518 PWID enrolled in services, and 795 kits of two 4 mg dose naloxone were distributed to participants. In addition to sterile needles and injection supplies, IDEA-SSP participants are provided with education on safer injection practices. This education includes instruction on the use of tester shots, using drugs with trusted friends, awareness of locations of previous overdoses, and unusually potent or otherwise toxic effects of commonly used drugs [7].

Importantly, SSP services include community distribution of take-home naloxone kits [8]. Take-home naloxone is an effective strategy for mitigating poor overdose outcomes as it reduces the time to administration versus activation of emergency medical services [9]. Community naloxone distribution removes barriers to naloxone access, a critical feature for populations that experience significant hesitation when seeking medical care, partially due to uninsured status, systemic bias, and stigma associated with drug use. PWID are often first responders to overdoses and reverse an overwhelming majority of community overdoses. A national survey from 1996 to 2014 reported over 26,400 overdose reversals with PWID conducting 82.8% of reversals [10]. Other research shows that PWID deploy take-home naloxone nearly ten times as frequently versus laypersons who do not use drugs—emphasizing the need to prioritize PWID in naloxone distribution efforts [11].

Multiple systematic reviews have found take-home naloxone programs to be both safe and effective, leading to increased survival rates among participants as well as decreases in community overdose mortality rates [12–14]. Although systematic analyses have found take-home naloxone programs are effective in reducing overdose deaths among participants, few studies assess the impact of take-home naloxone programs on hospitalizations [13–15]. We present a study analyzing early effects of the IDEA-SSP on the incidence of opioid overdose-associated admissions at a county safety-net hospital in south Florida.

## Methods

We conducted a 25-month retrospective review of hospitalized patients’ data at Jackson Memorial Hospital (JMH), a public hospital in Miami, Florida, that serves people without regard for insurance status. The period of review encompassed December 1, 2015, to January 1, 2018. JMH is the only safety-net hospital in Miami-Dade County and is within a half-mile proximity of the IDEA-SSP. Data were separated into two periods, with December 1, 2016—the establishment of the IDEA-SSP—as an index date. To increase the specificity of the query, data from December 1, 2016, to January 1, 2017, was excluded to allow time for sufficient community enrollment. An algorithm used by Tookes et al. was adapted using International Classification of Diseases, Tenth Revision (ICD-10) codes to query the JMH electronic discharge and billing records for patients aged 18–85 (see Supplemental Table 1) [16]. A combination of ICD-10 codes for opioid use and injection-related infections (IRI) was used to identify people who inject opioids (PWIO). Opioid codes included ICD-10 diagnoses related to opioids (see Supplemental Table 2). IRI included endocarditis, bacteremia/sepsis, osteomyelitis, abscesses, and/or cellulitis diagnoses.

**Fig. 1:**
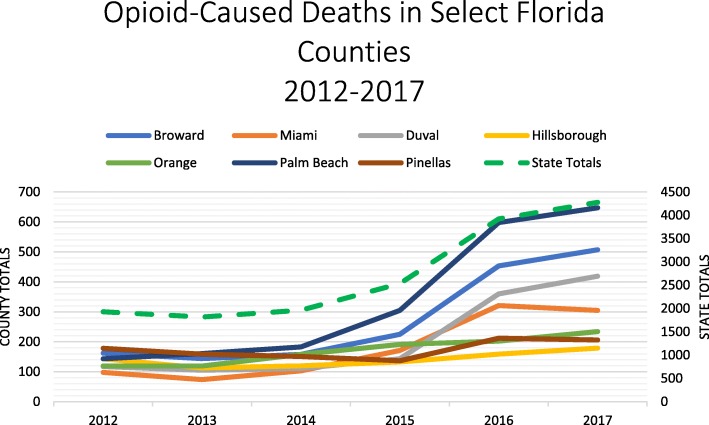
Opioid-caused deaths in select Florida counties 2012–2017

Medical records were abstracted for demographic information, length of stay (LOS), insurance status, and discharge status. Additionally, we independently analyzed publicly available Florida Department of Law Enforcement Medical Examiner Commission reports from 2012 to 2017 to identify regional and statewide trends in opioid-related mortality to compare to local findings (Fig. 1).

## Analysis

Descriptive statistics and frequency distributions for demographics, insurance status, and hospital use variables were utilized. Hospital use variables included discharge status and LOS for each hospitalization. Categorical data were described with numbers and percentages. Comparisons between pre- and post-index in frequencies of clinical and social demographic characteristics were analyzed by chi-square or Fisher’s exact test. The chi-square test and Fisher’s exact test can assess for independence between two variables when the comparing groups are independent and not correlated. Fisher’s exact test was used for the analysis of demographic factors including race, age in year, and insurance status. Chi-square was used for the analysis of PWID overdose-associated admissions. Because some continuous variables, such as age and LOS, were not normally distributed, the Wilcoxon rank-sum test was used for the comparisons. The results were reported as median and interquartile range. All analyses were performed in SAS 9.4 (SAS Institute Inc., Cary, NC).

## Results

### Demographics

Three hundred two PWIO admissions were identified: 146 pre-index vs. 156 post-index (*p* = 0.12) (Table 1). Race, sex, age, and insurance did not differ across pre- and post-index cohorts. Only 3% of PWIO had private insurance across the 2-year timespan. Approximately 60% of PWIO were uninsured, with no significant difference between cohorts (*p* = 0.88). Hospital mortality rates were not significantly different between the pre- and post-index cohorts. Nine (3%) patients died during the hospital stay as determined by an “expired” discharge status: five pre-index vs. four post-index (*p* = 0.74).

**Table 1:** PWIO demographics

	Pre-index, *n* = 146; # (%)	Post-index, *n* = 156; # (%)	*p* value
**Biological sex**			**0.46**
Male	103 (70.6)	103 (66.0)	
Female	43 (29.5)	53 (34.0)	
**Race**			**0.64**
White	118 (80.8)	124 (79.5)	
Black	27 (18.5)	29 (18.6)	
Others	1 (0.30)	3 (1.9)	
**Ethnicity**			
Hispanic	65 (44.5)	57 (36.5)	**0.16**
Non-Hispanic	81 (55.5)	99 (63.5)	
**Age in years**			**0.17**
16–29	22 (15.1)	30 (19.2)	
30–39	47 (32.2)	47 (30.1)	
40–49	36 (24.7)	48 (30.8)	
50–59	30 (20.6)	20 (12.8)	
60–65	9 (6.2)	5 (3.2)	
65–85	2 (1.4)	6 (3.9)	
**Insurance status**			**0.90**
Uninsured	85 (58.2)	95 (60.9)	
Medicaid	31 (21.2)	34 (21.8)	
Medicare + Federal	24 (16.4)	21 (13.5)	
Private	5 (3.4)	4 (2.6)	
Others	1 (0.7)	2 (1.3)	
**Median length of stay**	4	2	**0.14**
**Expired during study period**	5 (3.4)	4 (2.6)	**0.74**
**PWIO overdose-associated admissions**	20 (13.7)	6 (3.9)	**0.0034**

### Overdose sequela

Overdose-associated admissions significantly changed in the post-index cohort vs. the pre-index cohort. In the pre-index cohort, 14% of admissions involved an overdose diagnosis, vs. 4% in the post-index cohort (*p* = 0.0034).

### Florida opioid-caused deaths

State medical examiner findings demonstrated overall increasing opioid-related deaths in Florida between 2010 and 2017. From 2014 to 2016, opioid-caused deaths increased. The rate of increase declined from 2016 to 2017 except in Pinellas and Miami-Dade counties, where opioid-related mortality decreased.

## Discussion

Opioid-caused deaths increased in Florida following legislative efforts to close “pill mills” in 2012, with resulting increases seen in counterfeit opioid pills and heroin use [15]. This data explores opioid epidemic-related morbidity and mortality in south Florida through the lens of hospital admissions following the implementation of IDEA-SSP. With the introduction of fentanyl and high-potency analogues into the drug supply, sharp increases in opioid mortality were seen statewide between 2014 and 2016 [2, 17]. Given the heretofore unmitigated statewide overdose crisis, it would be expected that hospital data would reflect regional trends of increasing overdose-associated admissions. However, following SSP implementation, while the number of PWIO in our cohort did not change significantly, overdoses reported in PWIO decreased significantly. The temporal association suggests that the IDEA-SSP community distribution of take-home naloxone may have produced early effects in mitigating overdose-associated morbidity and mortality.

Several statewide opioid epidemic interventions were implemented before and directly following the study period, including a concerted law enforcement effort to close “pill mills” [17]. However, these statewide policies should theoretically affect all counties equally and thus do not temporally explain Miami-Dade’s decline in overdose deaths as reported by the Florida Department of Law Enforcement Medical Examiners Commission (Fig. 1). During the study period, the IDEA-SSP distributed 795 naloxone kits to participants and 387 reversals were reported. Between 2016 and 2017, opioid-related mortality in Miami-Dade County declined 5%, from 321 deaths to 305 deaths. Similar declines were not seen in neighboring counties. Considered together, these data suggest early impacts of the first legal SSP in the state, operating in Miami-Dade County.

More low-barrier SSPs are needed across Florida to increase naloxone access among PWID and reduce statewide opioid-related morbidity and mortality. Due to negative experiences PWID have when receiving services in traditional health care settings, they may be less likely to visit such settings to access naloxone, highlighting the importance of establishing naloxone distribution programs in low-barrier settings where PWID may feel more comfortable—namely SSPs and other harm reduction modalities. Recent modeling simulating the impact of 13 naloxone distribution modalities on overdose deaths estimated expanding naloxone distribution through a single SSP can reduce a community’s overdose deaths by 65% [18].

Limitations to this study exist. The ICD-10 does not have diagnosis codes for injection drug use or sequelae. This study relied on a novel ICD-10 adaptation of an ICD-9-based algorithm using codes for drug use and infectious consequences [16]. Additionally, the stigma associated with injection drug use remains widespread, and patients may not have reported use, resulting in under-documentation. Most importantly, our data do not imply causality between the establishment of the SSP and the decrease in opioid-associated admissions. Previous epidemiologic evaluations of SSPs describe lag times between community SSP implementation and decline in chronic infections [19]. An analysis of HIV rates among PWID in Baltimore only noted a significant decline after 5 years of increasing SSP service coverage, with sustained decline demonstrated thereafter [19]. Future research should explore longitudinal effects of the IDEA-SSP.

Despite these limitations, this study reveals a significant decrease in overdose-associated admissions among PWIO at a county safety-net hospital following the implementation of the IDEA-SSP in the setting of the contemporary Florida overdose crisis. Taken alongside medical examiner data, this study demonstrates trends of decreasing opioid overdose-related morbidity and mortality in Miami-Dade County. SSPs and take-home naloxone may impact the number of overdose-associated hospital admissions and warrant further study.

## Supplementary information

**Additional file 1: Supplementary Table 1.** JMH PWIO. This data consists of the 302 admission of people who inject opioids that we analyzed in this manuscript.

**Additional file 2: Supplemental Table 2.** JMH PWIO ICD-10 Codes. This table contains the complete list of ICD-10 codes used for inclusion in the study as described in the Methods section.

## Data Availability

The de-identified dataset is available as Supplementary Table 1. Florida Department of Law Enforcement Medical Examiners Commission Reports are publicly available data.

## Abbreviations

IDEA-SSP: University of Miami IDEA Syringe Services Program
PWID: People who inject drugs
JMH: Jackson Memorial Hospital
ICD-10: International Classification of Diseases, Tenth Revision
IRI: Injection-related infections
PWIO: People who inject opioids
LOS: Length of stay

## References

[1] Scholl L, Seth P, Kariisa M, Wilson N, Baldwin G (2018). Drug and opioid-involved overdose deaths - United States, 2013-2017. MMWR Morb Mortal Wkly Rep..

[2] Commission ME (2017). Drug identified in deceased persons by Florida medical examiners.

[3] Centers for Disease Control and Prevention. HIV surveillance report, 2018 (Preliminary); vol. 30. http://www.cdc.gov/hiv/library/reports/hiv-surveillance.html. Published November 2019. Accessed [1/31/2020].

[4] Alex Wodak AC, World Health Organization (2004). Effectiveness of sterile needle and syringe programming in reducing HIV/AIDS among injecting drug Switzerland: World Health Organization.

[5] Centers for Disease Control and Prevention. Summary of information on the safety and effectiveness of Syringe Services Programs (SSPs) [Available from: https://www.cdc.gov/ssp/syringe-services-programs-summary.html. Accessed 1 Oct 2019.

[6] AIDs UN. Do no harm - health, human rights and people who use drugs. 2016 April 15 2016.

[7] Mars SG, Ondocsin J, Ciccarone D (2018). Toots, tastes and tester shots: user accounts of drug sampling methods for gauging heroin potency. Harm Reduct J..

[8] Reed M, Wagner KD, Tran NK, Brady KA, Shinefeld J, Roth A (2019). Prevalence and correlates of carrying naloxone among a community-based sample of opioid-using people who inject drugs. Int J Drug Policy..

[9] Chimbar L, Moleta Y (2018). Naloxone effectiveness: a systematic review. J Addict Nurs..

[10] Wheeler E, Jones TS, Gilbert MK, Davidson PJ. Centers for Disease Control and Prevention. Opioid overdose prevention programs providing naloxone to laypersons - United States, 2014. MMWR Morbidity and mortality weekly report. 2015;64(23):631–5. PMC4584734 26086633

[11] Bennett AS, Bell A, Doe-Simkins M, Elliott L, Pouget E, Davis C (2018). From peers to lay bystanders: findings from a decade of naloxone distribution in Pittsburgh. PA. J Psychoactive Drugs..

[12] Giglio RE, Li G, DiMaggio CJ (2015). Effectiveness of bystander naloxone administration and overdose education programs: a meta-analysis. Inj Epidemiol.

[13] McDonald R, Strang J (2016). Are take-home naloxone programmes effective? Systematic review utilizing application of the Bradford Hill criteria. Addiction..

[14] Olsen A, McDonald D, Lenton S, Dietze PM (2018). Assessing causality in drug policy analyses: How useful are the Bradford Hill criteria in analysing take-home naloxone programs?. Drug Alcohol Rev..

[15] Alvarez L. Florida shutting ‘pill mill’ clinics. The New York Times. 2011.

[16] Tookes H, Diaz C, Li H, Khalid R, Doblecki-Lewis S (2015). A cost analysis hospitalizations for infections related to injection drug use at a county safety-net hospital in Miami. Florida. PLoS One..

[17] Rutkow L, Chang H-Y, Daubresse M, Webster DW, Stuart EA, Alexander GC (2015). Effect of Florida’s prescription drug monitoring program and pill mill laws on opioid prescribing and use. JAMA Internal Medicine..

[18] Keane C, Egan JE, Hawk M (2018). Effects of naloxone distribution to likely bystanders: results of an agent-based model. Int J Drug Policy..

[19] BCHD. Jurisdictional plan for HIV prevention in Baltimore City. Baltimore City; 2012. 2015.

