# Supplementary material for: Hospital admissions among people who inject opioids following syringe services program implementation
Source: Harm Reduct J. 2020 May 12;17:30. doi: 10.1186/s12954-020-00376-1 (PMC7216361; doi:10.1186/s12954-020-00376-1)
Supplement: Supplementary file 2 — Additional file 2: Supplemental Table 2. JMH PWIO ICD-10 Codes. This table contains the complete list of ICD-10 codes used for inclusion in the study as described in the Methods section. [file 12954_2020_376_MOESM2_ESM.docx]

**Supplemental Table 2: JMH PWIO ICD-10 Codes**

| **Drug Abuse Diagnoses** | **Drug Abuse Diagnoses** |
| --- | --- |
| Opioids | F11, F11.1, F11.10, F11.11, F11.12, F11.120, F11.121, F11.122, F11.129, F11.14, F11.15, F11.150, F11.151, F11.159, F11.18, F11.181, F11.182, F11.188, F11.19, F11.2, F11.20, F11.22, F11.220, F11.221, F11.222, F11.229, F11.23, F11.24, F11.25, F11.250, F11.251, F11.29, F11.259, F11.28, F11.281, F11.282, F11.288, F11.9, F11.92, F11.920 F11.921, F11.922, F11.929, F11.93, F11.94, F11.95, F11.950, F11.951, F11.959, F11.98, F11.981, F11.982, F11.988, F11.99 |
| **Infections** |  |
| Endocarditis | I38, I33.9, I33.0, I39, B37.6 |
| Sepsis/  Bacteremia | A41.2, A41.01, A41.1, A41.02, A41.51, A40.3, A41.4, A41.50, A41.3, A41.52, A41.53, A41.59, A41.89, I26.90, I40.0, I76, R65.21, R78.81, R65.20, A41.9, A40.9, B37.7, R57.8 A42.7 |
| Osteomyelitis | H05.021, H05.022, H05.023, H05.029, M46.20, M46.21, M46.22, M46.23, M46.24, M46.25, M46.26, M46.27, M46.28, M86.00, M86.011, M86.012, M86.019, M86.021, M86.022, M86.029, M86.031, M86.032, M86.039, M86.041, M86.042, M86.049, M86.051, M86.052, M86.059, M86.061, M86.062, M86.069, M86.071, M86.072, M86.079, M86.08, M86.09, M86.10, M86.111, M86.112, M86.119, M86.121, M86.122, M86.129, M86.131, M86.132, M86.139, M86.141, M86.142, M86.149, M86.151, M86.152, M86.159, M86.161, M86.162, M86.169, M86.171, M86.172, M86.179, M86.18, M86.19, M86.20, M86.211, M86.212, M86.219, M86.221, M86.222, M86.229, M86.231, M86.232, M86.239, M86.241, M86.242, M86.249, M86.251, M86.252 M86.259, M86.261, M86.262, M86.269, M86.271, M86.272, M86.279, M86.28, M86.29, M86.30, M86.311, M86.312, M86.319, M86.321, M86.322, M86.329, M86.331, M86.332, M86.339, M86.341, M86.342, M86.349, M86.351, M86.352, M86.359, M86.361, M86.362, M86.369, M86.371, M86.372, M86.379 M86.38, M86.39, M86.40, M86.411, M86.412, M86.419, M86.421 M86.422, M86.429, M86.431, M86.432, M86.439, M86.441, M86.442, M86.449, M86.451, M86.452, M86.459, M86.461, M86.462, M86.469, M86.471, M86.472, M86.479, M86.48, M86.49 M86.50, M86.511, M86.512, M86.519, M86.521,M86.522, M86.529, M86.531, M86.532, M86.539, M86.541, M86.542, M86.549, M86.551, M86.552, M86.559, M86.561, M86.562, M86.569, M86.571, M86.572, M86.579, M86.58, M86.59, M86.60, M86.611, M86.612, M86.619, M86.621, M86.622, M86.629, M86.631, M86.632, M86.639, M86.641, M86.642, M86.649, M86.651, M86.652, M86.659, M86.661, M86.662, M86.669, M86.671, M86.672, M86.679, M86.68, M86.69, M86.8X0, M86.8X1 M86.8X2, M86.8X3, M86.8X4, M86.8X5, M86.8X6, M86.8X7, M86.8X8, M86.8X9, M86.9, G04.89 G04.91, G05.4 |
| Skin and Soft Tissue Infection | L03, L03.0, L03.01, L03.011, L03.012, L03.019, L03.02, L03.021, L03.022, L03.029, L03.03 L03.031, L03.032, L03.04, L03.041, L03.042, L03.049, L03.1, L03.11, L03.111, L03.112, L03.113 L03.114, L03.115, L03.116, L03.119, L03.12, L03.121, L03.122, L03.123, L03.124, L03.125, L03.126, L03.129, L03.221, L03.222, L03.8, L03.81, L03.811, L03.818, L03.89, L03.891, L03.898, L03.9, L03.90, L03.91, G06.0, G06.1, G06.2, G07, G09, K65.1, K68.12, K68.19, K75.0, L03.039, L03.319, L03.329, L03.317, L98.8, M72.6, K63.0 |
